# 中国复发难治性急性髓系白血病诊疗指南（2021年版）

**DOI:** 10.3760/cma.j.issn.0253-2727.2021.08.002

**Published:** 2021-08

**Authors:** 中华医学会血液学分会白血病淋巴瘤学组

**修订要点：**

• 增加推荐等级，证据等级按牛津大学EBM中心（Oxford Centre for Evidence-based Medicine Levels of Evidence）关于文献类型的五级标准。

• 将治疗原则的“新的靶向治疗药物”前移至第一位，突出了其重要地位；删除了原来的“使用耐药逆转剂”。

• 强调复发难治性急性髓系白血病（AML）患者应重新进行染色体和分子遗传学的检查（如二代测序、RNA测序等）以帮助选择合适的治疗方案或临床试验。

• 增加了靶向药物吉瑞替尼、索拉菲尼治疗FLT3突变患者，并指出两种药物所用于的突变类型。增加了BCL2抑制剂维奈克拉的用法、剂量以及联合用药的药物。

• 增加了对于原发耐药或早期复发且无法缓解的患者也可以直接采取异基因造血干细胞移植作为挽救治疗措施的内容。

一、复发、难治性AML诊断标准

1. 复发性AML诊断标准：AML完全缓解（CR）后外周血再次出现白血病细胞或骨髓中原始细胞≥5％（除外巩固化疗后骨髓再生等其他原因）或髓外出现白血病细胞浸润。

2. 难治性白血病诊断标准：经过标准方案治疗2个疗程无效的初治病例；CR后经过巩固强化治疗，12个月内复发者；在12个月后复发但经过常规化疗无效者；2次或多次复发者；髓外白血病持续存在者。

二、复发、难治性AML预后评估及治疗策略

对于复发的年轻AML患者（年龄≤60岁），欧洲白血病网（ELN）推荐根据患者年龄、缓解至复发的时间、细胞遗传学以及是否接受过造血干细胞移植（HSCT）积分系统进行预后评估（表1）。

**表1:** 年轻复发急性髓系白血病（AML）预后评估

预后组别	积分	1年OS率（％）	5年OS率（％）
低危（占9％）	0～6	70	46
中危（占25％）	7～9	49	18
高危（占66％）	10～14	16	4

注：OS：总生存。评估适用于15～60岁复发的AML（除外M_3_），分数计算依据如下：缓解至复发时间：>18个月计0分，7～18个月计3分，≤6个月计5分；初发时细胞遗传学：inv（16）或t（16;16）计0分；t（8;21）计3分；其他计5分；是否进行过造血干细胞移植：否计0分，是计2分；复发时年龄：≤35岁计0分，36～45岁计1分，>45岁计2分。分数累加后得出复发患者的积分，并归为低危、中危或高危，可作为参考

近年来出现不少AML的治疗新药，这类新药为一些复发难治AML患者带来再次获得缓解的机会。即便如此，复发难治白血病的疗效依然欠佳。对于复发难治AML患者应该再次做染色体及二代基因突变检测，以明确是否存在或新出现某些特殊异常染色体与突变基因，为再次治疗方案选择提供帮助。

难治性白血病的治疗原则包括：①新的靶向治疗药物；②中、大剂量的阿糖胞苷（Ara-C）组成的联合方案；③使用无交叉耐药的新药组成的新的联合化疗方案；④异基因造血干细胞移植（allo-HSCT）；⑤免疫治疗。

三、复发、难治性AML治疗建议

在选择化疗方案时，应综合考虑患者细胞分子遗传学、突变基因、复发时间、患者个体因素（如年龄、体能状况、合并症、早期治疗方案）等因素，以及患者及家属的治疗意愿。再次强调对复发难治性AML患者应重新进行染色体和分子遗传学的检查（如二代测序、RNA测序等）以帮助选择合适的治疗方案或临床试验。除特殊说明外，以下证据等级均为2a。针对靶向药物的使用，除了用作维持治疗，一般不主张单药使用，大多联合去甲基化药物或与化疗药物联合。复发难治AML的治疗原则：按照年龄及体能状况、复发时间、突变基因进行分层。

（一）年龄<60岁

早期复发者（缓解后12个月以内复发者）：①临床试验（强烈推荐）；②靶向药物治疗，如有FLT3突变的患者可以选择吉瑞替尼、索拉菲尼等。其他可根据情况选用BCL2抑制剂维奈克拉、IDH1抑制剂艾伏尼布、IDH2抑制剂恩西地平等；③挽救化疗，获得CR后继而行同胞相合或无关供者HSCT；④直接进行allo-HSCT。

晚期复发者（缓解12个月以上复发者）：①重复初始有效的诱导化疗方案，如达到再次缓解，考虑进行allo-HSCT；②临床试验；③靶向药物治疗；④挽救化疗，CR后行同胞相合或无关供者HSCT。

难治性患者按照早期复发者方案处理。

（二）年龄≥60岁

早期复发者：①临床试验（强烈推荐）；②新药（包括靶向药物与非靶向药物）治疗；③最佳支持治疗；④挽救化疗，CR后如体能状况好可以考虑进行allo-HSCT。

晚期复发者：①临床试验（强烈推荐）；②重复初始有效的诱导化疗方案；③新药（包括靶向药物与非靶向药物）治疗；④挽救化疗，CR后如体能状况好可以考虑allo-HSCT；⑤最佳支持治疗（用于不能耐受或不愿意进一步治疗的患者）。

四、复发难治性AML的治疗方案

（一）靶向治疗±去甲基化药物

1. FLT3-ITD突变：

（1）吉瑞替尼（Gilteritinib）：是一种新型、强效、高选择性、Ⅰ型口服FLT3/AXL抑制剂，与Ⅱ型抑制剂的不同在于吉瑞替尼通常不受激活环突变（如D835点突变）的影响，能够结合FLT3突变的活性构象和非活性构象。可用于FLT3-ITD和FLT3-TKD突变。治疗剂量为120 mg/d（证据等级1a）[1]–[2]。

（2）索拉菲尼+去甲基化药物（阿扎胞苷或地西他滨）：索拉菲尼200 mg，每日2次；阿扎胞苷75 mg/m^2^，第1～7天；或地西他滨20 mg/m^2^，第1～5天（证据等级2a）。

2. FLT3-TKD突变：吉瑞替尼，治疗剂量为120 mg/d（证据等级1a）[2]。

3. IDH1突变：艾伏尼布500 mg每日1次，可联合去甲基化药物，去甲基化药物剂量及用法同上（证据等级2a）[4]–[5]。

4. IDH2突变：恩西地平100 mg每日1次，可联合去甲基化药物，去甲基化药物剂量及用法同上（证据等级2a）[3]。

5. 由于IDH1/2突变对BCL2抑制剂维奈克拉均比较敏感，因此IDH1/2突变者可以应用维奈克拉联合去甲基化药物（证据级别2a）。

（二）联合化疗

分为强烈化疗和非强烈化疗。强烈化疗方案包含以嘌呤类似物（如氟达拉滨、克拉屈滨）为主的方案，缓解率为30％～45％，中位生存期8～9个月。非强烈化疗方案包括去甲基化药物、小剂量Ara-C和BCL2抑制剂等。

1. 强烈化疗方案：用于一般情况好，耐受性好的患者。

（1）CLAG±IDA/Mitox方案：克拉屈滨、Ara-C、G-CSF，加或不加去甲氧柔红霉素（IDA）/米托蒽醌（Mitox）。具体用法：克拉屈滨5 mg/m^2^，第1～5天；Ara-C 1～2 g/m^2^，克拉屈滨用后4 h使用，第1～5天，静脉滴注3 h；G-CSF 300 µg/m^2^，第0～5天（WBC>20×10^9^/L暂停）；IDA 10～12 mg/m^2^，第1～3天或Mitox 10～12 mg/m^2^，第1～3天（证据等级2a）。

（2）大剂量Ara-C±蒽环类药物：Ara-C 1～3 g/m^2^，每12 h 1次，第1、3、5天；联合DNR 45 mg/m^2^或IDA 10 mg/m^2^，第2、4、6天。或Ara-C（既往没有用过大剂量Ara-C的患者可以选择）3 g/m^2^，每12 h1次，第1～3天（证据等级2a）[8]。

（3）FLAG±IDA方案：氟达拉滨、Ara-C、G-CSF±IDA。具体用法：氟达拉滨30 mg/m^2^，第1～5天；Ara-C 1～2 g/m^2^，氟达拉滨用后4 h使用，第1～5天，静脉滴注3 h；G-CSF 300 µg/m^2^，第0～5天；IDA 10～12 mg/m^2^，第1～3天（证据等级2a）。

（4）HAA（HAD）方案：高三尖杉酯碱（HHT）、Ara-C、阿克拉霉素（Acla）或柔红霉素（DNR）。具体用法：HHT 2 mg/m^2^，第1～7天（或HHT 4 mg/m^2^，分2次给药，第1～3天）；Ara-C 100～200 mg/m^2^，第1～7天；Acla 20 mg/d，第1～7天（或DNR45 mg·m^−2^·d^−1^，第1～3天）[6]。

（5）EA±Mitox方案：依托泊苷（Vp16）、Ara-C ±Mitox。具体用法：Vp16 100 mg/m^2^，第1～5天；Ara-C 100～150 mg/m^2^，第1～7天；Mitox 10 mg/m^2^，第1～5天（证据等级2a）。

（6）CAG方案：G-CSF 300 µg/m^2^，每12 h 1次，第0～14天；Acla 20 mg/d，第1～4天；Ara-C 10 mg/m^2^，皮下注射，每12 h 1次，第1～14天（证据等级2a）。

2. 非强烈化疗方案：对于体能状况差、耐受较差的患者，可选择非强烈化疗方案。

（1）去甲基化药物（阿扎胞苷、地西他滨）：①阿扎胞苷75 mg/m^2^，第1～7天，28 d为1个疗程，直至患者出现疾病恶化或严重不良反应（证据等级2a）；②地西他滨20 mg/m^2^，第1～5天，28 d为1个疗程，直至患者出现疾病恶化或严重不良反应（证据等级2a）。

（2）小剂量Ara-C：Ara-C 10 mg/m^2^，皮下注射，每12 h 1次，第1～14天（证据等级2b）。

（3）维奈克拉+去甲基化药物/小剂量Ara-C：①维奈克拉联合去甲基化药物：维奈克拉剂量为第1天100 mg，第2天200 mg，第3天开始每天400 mg直至第28天；去甲基化药物：阿扎胞苷75 mg/m^2^，第1～7天；地西他滨25 mg/m^2^，第1～5天（证据等级2a）[7]–[9]；②维奈克拉联合小剂量Ara-C：维奈克拉剂量为第1天100 mg，第2天200 mg，第3天400 mg，第4天开始每天600 mg直至第28天；Ara-C 10 mg/m^2^，皮下注射，每12 h 1次，第1～10天（证据等级2a）。

（4）allo-HSCT：复发难治性AML患者获得缓解后如条件许可应尽早进行allo-HSCT。对于某些患者，尤其是原发耐药或早期复发且无法缓解的患者也可以直接采取allo-HSCT作为挽救治疗措施[10]。

（5）免疫治疗：主要包括CAR-T细胞治疗和双靶点抗体治疗。由于AML表面的特异性抗原不仅在白血病细胞膜上表达，同时也在正常造血干细胞膜上表达，CAR-T细胞治疗和双靶点抗体治疗可能会导致正常造血干细胞的损伤，目前尚处于试验阶段。
